# 

**Published:** 1874-09-15

**Authors:** 


### *NEW BOOKS RECEIVED.*

THE PHYSIOLOGY OF MAN.—Designed to represent the existing state of Physiological Science, as applied tc the function of the human body. By Austin Flint, Jr., M.D. Vol. V., *Special Senses, Generation.* New York: D. Appleton & Co. For sale by W. B. Keen, Cooke & Co., Chicago.

---

THE MEDICAL REGISTER AND DIRECTORY OF THE UNITED STATES.—Systematically arranged by States. Office of Medical and Surgical Reporter, Philadelphia, Pa.

---

THE NEW MODIFIED CAMMAN STETHOSCOPE.—The modified Camman Stethoscope, of which we published a cut and a short description in a recent number of the EXAMINER, is now manufactured by Messrs. Tieman, of New York, and for sale by their agents, Messrs. Bliss & Torrey, of this city. These instruments have the Flint curve to the tubes, and in general style and finish are perfect.

This form of Stethoscope is also manufactured by Messrs. Codman & Shurtleff, of Boston, and for sale by their various agencies.

